# Implant Placement in Patients under Treatment with Rivaroxaban: A Retrospective Clinical Study

**DOI:** 10.3390/ijerph17124607

**Published:** 2020-06-26

**Authors:** Guido Galletti, Fortunato Alfonsi, Angelo Raffaele, Nicola Alberto Valente, Sibylle Chatelain, Roni Kolerman, Chiara Cinquini, Stefano Romeggio, Giovanna Iezzi, Antonio Barone

**Affiliations:** 1 Department of Surgical, Medical, Molecular and of Critical Area Pathologies, Complex Operative Unit of Stomatology and Oral Surgery, University-Hospital of Pisa, University of Pisa, 56126 Pisa, Italy; studiodralfonsi@gmail.com (F.A.); chiara.cinquini@gmail.com (C.C.); barosurg@gmail.com (A.B.); 2 Complex Operative Unit of Geriatrics, SS Filippo e Nicola Hospital, 67051 Avezzano, Italy; araffaele@asl1abruzzo.it; 3 Unit of Oral Surgery and Implantology, Service of Maxillofacial and Buccal Surgery, Department of Surgery, Geneva University Hospitals, University of Geneva, 1205 Geneva, Switzerland; navalentedds@gmail.com (N.A.V.); chatelainsibylle@gmail.com (S.C.); 4 Department of Stomatology, Faculty of Dentistry, University of Seville, 41009 Seville, Spain; 5 Department of Oral and Maxillofacial Surgery, Vaud University Hospital (CHUV Centre Hospitalier Universitaire Vaudois), 1011 Lausanne, Switzerland; 6 Department of Periodontology and Dental Implantology, The Maurice and Gabriela Goldschleger School of Dental Medicine, Tel Aviv University, Tel Aviv 6997801, Israel; kolerman@netvision.net.il; 7 Private Practice, Innova Clinique, 28845 Domodossola, Italy; romeggiostefano@yahoo.it; 8 Department of Medical, Oral and Biotechnological Sciences, School of Dentistry, University of Chieti and Pescara, 66100 Chieti, Italy; gio.iezzi@unich.it

**Keywords:** anticoagulants, implant placement, immediate loading

## Abstract

The management of patients under treatment with Direct Oral Anticoagulants (DOACs) has led clinicians to deal with two clinical issues, such as the hemorrhagic risk in case of non-interruption or the risk of thromboembolism in case of suspension of the treatment. The primary aim of this retrospective study was to evaluate the incidence of perioperative bleeding events and healing complications in patients who were under treatment with Rivaroxaban and who received dental implants and immediate prosthetic restoration. Patients treated with Rivaroxaban (Xarelto 20 mg daily) and who needed implant rehabilitation were selected. Four to six implants were placed in mandibular healed sites or fresh extraction sockets. All patients, in agreement with their physicians, interrupted the medication for 24 h and received implants and immediate restorations. Twelve patients and 57 implants were analyzed in the study. No major postoperative bleeding events were reported. Three patients (25%) presented slight immediate postoperative bleeding controlled with compression only. The implant and prosthetic survival rate were both 100% after 1 year. Within the limitations of this study, multiple implant placement with an immediate loading can be performed without any significant complication with a 24 h discontinuation of Rivaroxaban, in conjunction with the patient’s physician.

## 1. Introduction

Oral Anticoagulant medications (OAM) have been used successfully in the prevention of thrombotic diseases, caused by myocardial infarction, cardiovascular stroke, atrial fibrillation, placement of a mechanical heart-valve prosthesis or deep venous thromboembolism [1]. Vitamin K antagonists (VKAs), such as Warfarin, were used for decades for the treatment of such diseases; and in patients with contraindications for VKAs, antiplatelet medications were prescribed as an alternative [2]. Despite the wide use and application of these drugs, several downsides have been reported, such as significant food and drug interactions, a narrow therapeutic index, and frequent need for monitoring coagulation status [3,4]. According to scientific literature, the suspension of vitamin K antagonists in patients undergoing dental treatments is generally not recommended [5]. The American College of Chest Physicians (ACCP) recommended that clinicians should not interrupt VKAs for dental treatments, especially when INR values are lower than 3.5, and the use of local hemostatic agents is strongly recommended to prevent and treat future bleeding events [6].

In the past few years, a new generation of Direct Oral Anticoagulants (DOACs) has been released on the market. The following medications, belonging to the DOACs category, are prescribed for reducing the risk of thrombo-embolic events: Dabigatran etexilate (inhibitor of Thrombin), Rivaroxaban, Apixaban, and Edoxaban (inhibitors of Factor X activated). Compared to Warfarin, the DOACs have a particular interference with the coagulation cascade since they have a rapid onset, shorter half-life, and fewer food or/and drug interactions [7,8]. Moreover, their administration on a fixed-dose provides a stable anticoagulation effect and makes it possible to avoid regular laboratory monitoring (e.g., INR) [7,9,10].

Rivaroxaban is an oxazolidinone derivative that acts as a direct inhibitor of active Factor X (FXa) in the coagulation cascade. This medication blocks the transformation of prothrombin into thrombin, and thus, ultimately inhibits blood clot formation [10]. This medication has even been used in patients suffering from non-valvular atrial fibrillation and experiencing some other cardiovascular diseases or diabetes mellitus in order to prevent thromboembolic events [11]. Rivaroxaban has a rapid onset (~3 h) and a plasma half-life of 5.7–9.2 h. Oral bioavailability is 80–100%, and the liver metabolizes it and 66% is excreted in the urine [7,12]. Several guidelines have been proposed to manage patients in treatment with oral anticoagulants and in need of oral surgery procedures. Indeed, many debates [12,13] have been reported in the scientific literature on whether or not to interrupt Oral Anticoagulant Medications (OAM) in case of simple oral surgery procedures. In the end, what has been highlighted is that anticoagulation interruption could severely harm the medically compromised patients, in terms of risk of having embolic events.

Conversely, the risk of having bleeding events could be easily handled in case of non-invasive oral surgery procedures [7,14]. Several studies have investigated ways to minimize the risk of perioperative bleeding in patients under DOACs treatment in case of non-interruption or risk of thromboembolism in the event of suspension of treatment [12,15]. Recent studies show that the administration of Rivaroxaban in patients receiving up to 3 implants does not augment the incidence of prolonged bleeding because it directly inhibits the coagulation factors, ensuring a safer and more predictable response [12,15]. However, no studies are reporting more than three implant placements within the same surgical procedure. The lack of literature regarding multiple (more than 3) implant placements in patients in treatment with DOACs forces clinicians to be more cautious when the suspension of the medication is considered. In order to reduce the number of surgical interventions, and therefore, the risks of bleeding events, immediate implant placement following tooth extractions and immediate restoration can be a valuable option. This treatment strategy has been widely demonstrated to be an effective procedure for restoring function and esthetics, thereby satisfying patient expectations [16,17,18,19]. According to the European Academy of Cardiology, the suspension of DOACs is unnecessary for single tooth extraction [10,20]. However, it is debated if this strategy is required for multiple tooth extractions or implant placements. 

The primary aim of this retrospective study was to evaluate the incidence of postoperative bleeding events and healing complications in patients taking Rivaroxaban treated with immediate full-arch rehabilitation and peri-implant bone augmentation when needed. The secondary aim was to analyze the implant and prosthetic survival rate at 1-year follow-up.

## 2. Materials and Methods 

### 2.1. Patient Selection

The present investigation was designed as a retrospective clinical study based on data from patients recruited and treated between September 2015 and November 2018 for immediate full-arch rehabilitation at the Unit of Oral Surgery and Implantology, University-Hospital of Geneva, Switzerland, and at the Department of Surgical, Medical, Molecular and of Critical Area Pathologies, University of Pisa, Italy.

Preoperative, intraoperative, and postoperative clinical data were retrieved from the patient records.

All the surgical and prosthetic phases were performed by the same surgeon (A.B.), postoperative evaluations were assessed by two different surgeons, one for the Geneva Hospital (G.G.) and another one for the Pisa Hospital (F.A.).

This study was conducted in full accordance with the Declaration of Helsinki (as revised, amended, and clarified in its version of 2008). Moreover, this study was approved by the Geneva Committee for Ethical Research (ethical approval number 2017-01-556) and by the Regional Ethical Committee Area Vasta Nord Ovest (Protocol n. 15943, on 24/10/2019). 

### 2.2. Inclusion Criteria 

Patients were included in the study according to the following criteria:

- Patients who underwent dental implant treatment.
- Patients who had received treatment with Rivaroxaban (Xarelto^®^ 20 mg/day, dose taken at night) for at least six months before implant placement.
- Patients who needed full-arch implant-supported mandibular rehabilitation.
- Patients who had complete medical and dental data records.

### 2.3. Exclusion Criteria 

Patients were excluded from the study according to the following criteria:

- History of antecedents of bleeding episodes during oral surgical interventions.
- Uncontrolled diabetes.
- Metabolic bone disorders.
- Impaired kidney function.
- Radiation therapy of the head or neck region
- Current chemotherapy.
- Drug or alcohol abuse.
- Untreated periodontal disease.
- Incomplete demographic, medical, and implant data.

### 2.4. Data Collection

An implant was deemed as an implant failure when complaints from the patients and clinical signs had led to implant removal. In this case, it was considered a missed implant. 

All demographic and medical patient data were collected: gender, sex, smoking habits, bruxism, general health status, in the presence of systemic pathologies, time since diagnosis, medications intake, and length of treatment were considered. The medical history data collection was especially focused on the reason for anticoagulant treatment and the presence of co-morbidities such as hypertension, diabetes, hypercholesterolemia, asthma. Prothrombin time (PT), complete blood count, hemoglobin values, and renal function were also evaluated from general health record data. Special attention was given to possible intake of medications such as antidepressant, proton-pump inhibitors, statins, and immunosuppressive drugs. 

**Figure 1:**
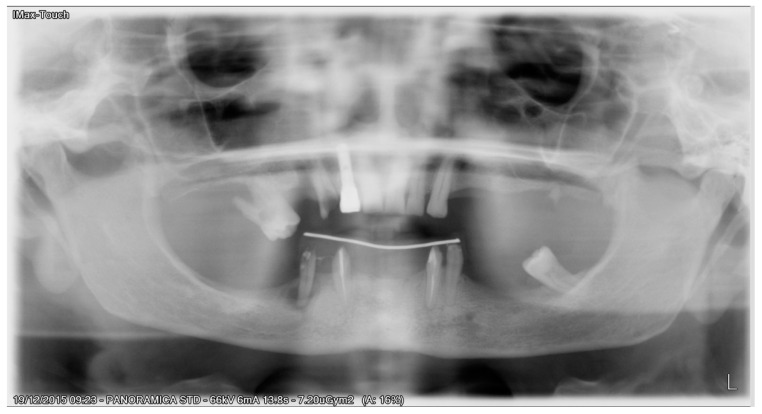
Preoperative panoramic X-ray.

All patients enrolled in this study had panoramic radiographs and/or cone-beam computed tomography (CBCT) (Figure 1). 

The implant treatment plan was established according to a diagnostic wax-up in order to evaluate occlusion, aesthetic parameters, and inter-maxillary relationship. On the base of this setup, a cross-arch provisional template and a surgical custom guide were prepared. Moreover, all patients received, before going through implant surgery, one or two sessions of oral hygiene instructions and periodontal non-surgical treatment to reduce the degree of inflammation in the whole mouth and especially for those sites that had to undergo tooth extraction and immediate implant placement. In agreement with the patient’s physician, all patients were instructed to interrupt their daily dose of Rivaroxaban the day before the surgery and to restart it the same day of the surgery. All the procedures were initiated in the early morning and finished after a couple of hours at most. On the day of surgery before starting any procedure, all patients underwent blood pressure, heart pulse rate, and oxygen saturation controls.

The patients received prophylactic antibiotic therapy of 2 g of amoxicillin (or 600 mg of clindamycin if allergic to penicillin) 1 h before the tooth extraction procedure and continued to take antibiotics postoperatively (1 g amoxicillin or 300 mg clindamycin, twice a day, for 5 days).

**Figure 2:**
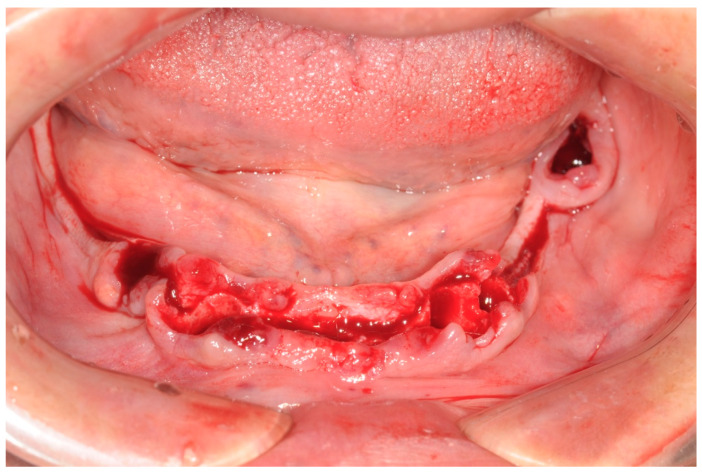
Operative site after tooth extractions.

All patients were treated under local anesthesia, and patients received four, five, or six implants in the mandible according to the prosthetic treatment plan. In the case of tooth extraction sites, a periodontal probe was used to assess the integrity of the extraction socket’s bony walls and of the adjacent bone peaks to evaluate the feasibility of an immediate implant (Figure 2). 

**Figure 3:**
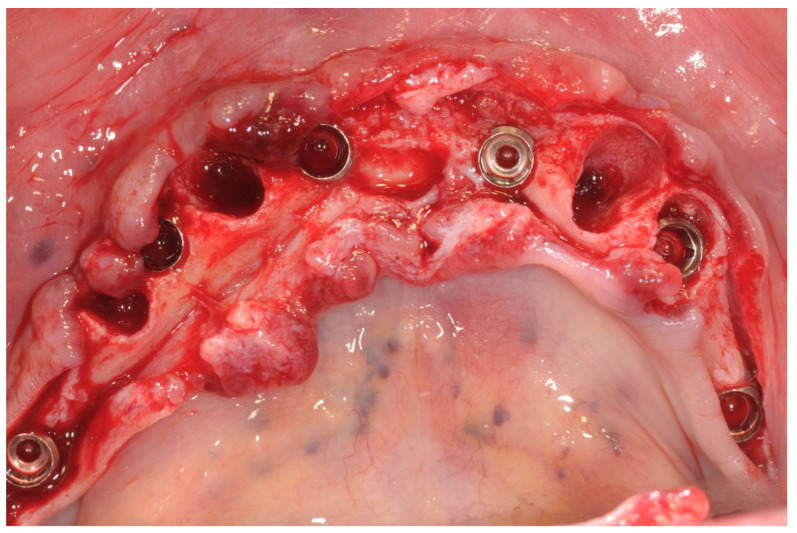
Implants were inserted, and flat abutment connected.

All patients received implants with a grit-blasted and acid-etched surface (Ossean^®^; Intra-Lock International^®^, Inc., Boca Raton, FL, USA) that were placed using a sterile surgical technique, as recommended by the manufacturer. Maximum care was taken to place the implants, regardless of whether they were inserted in an edentulous ridge or an extraction site, with a minimum insertion torque of 35 Ncm and not exceeding 50 Ncm (Figure 3). 

**Figure 4:**
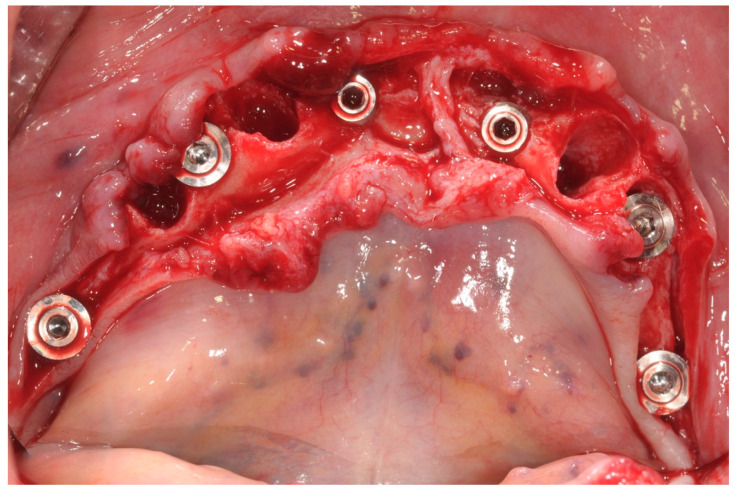
Flat abutment connection.

The peri-implant bone defects in the extraction sockets were grafted with a cortico-cancellous porcine bone (GTO, Tecnoss-Dental, Giaveno, Italy). Flat abutments (FlatOne^®^; Intra-Lock International^®^, Inc., Boca Raton, FL, USA) were then connected to the implants, and the flap was sutured (Figure 4). 

**Figure 5:**
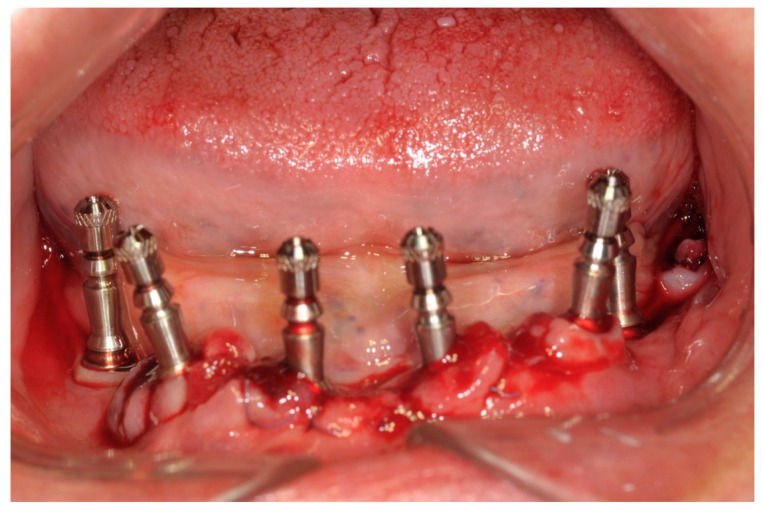
Transfer abutments before impressions.

After the surgical procedure, impressions were taken using a polyether elastomeric material (Impregum Penta^®^; 3M ESPE^®^, Milan, Italy) (Figure 5). 

Subsequently, the full-arch screw-retained prosthesis was finalized and delivered within 48 h after surgery.

**Figure 6:**
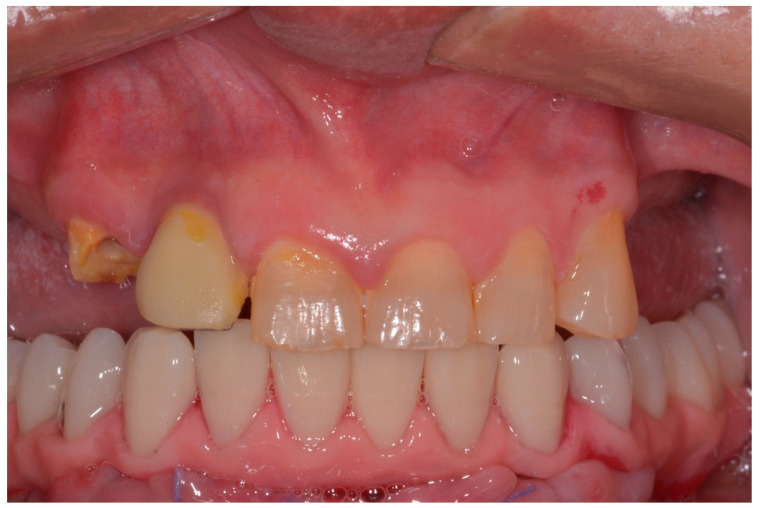
7 days postoperative control.

After completing the surgical procedures, but before being discharged, all patients were kept under control in the output clinic, compressing the surgically treated area with gauze for 30 min. If some bleeding was still present after 30 min, a compression with new gauzes and tranexamic acid was performed for an additional 30 min period. For those patients who still had some bleeding, local treatment with electrocauterization and additional sutures was performed. All patients were prescribed paracetamol 1000 mg tablets as a pain killer to be taken three times a day, as long as required. All patients were instructed to apply external ice packs to the surgical area for 12 h postoperatively. They were advised to avoid mouthwashes for the first 24 h after surgery. The sutures were removed 7 days after surgery (Figure 6).

On the day of immediate restoration delivery, the healing abutments were removed, the immediate restoration was inserted, and the abutment screws were tightened. Subsequently, the occlusion was carefully checked. All provisional prostheses were screw-retained and fabricated with a metal framework and resin. 

**Figure 7:**
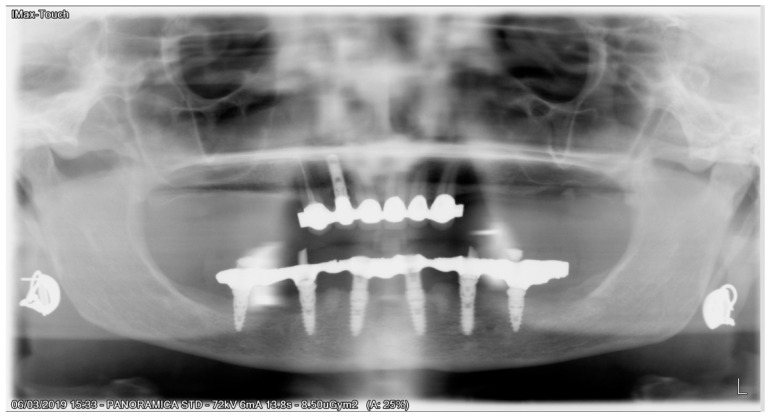
2 years postoperative panoramic X-ray.

Six months after surgery, all temporary restorations were replaced with final prosthetic restorations (Figure 7).

### 2.5. Outcome Variables

- Intraoperative bleeding evaluation, according to Bacci (2016) [21] over the seven-day period following implant insertion:
  ✓ No bleeding.
  ✓ Slight bleeding understood as slight oozing from the wound incision controlled with compressive gauze only.
  ✓ Moderate bleeding understood as large clots disrupting the surgical area and requiring additional hemostatic measures.
  ✓ Severe hemorrhaging, requiring major medical management.
- Evaluation of postoperative complications- such as edema, swelling, and hematoma- during 14 days following implant insertion;
- Implant survival rate at a 1-year follow-up evaluated based on the Albrektsson criteria [22].
- Prosthetic survival rate at a 1-year follow-up evaluated based on the loss of the prosthesis due to implant failure or its replacement for any other reason.

## 3. Results

Medical data records from a total of 15 patients were collected, two patients were excluded because implant data were not complete, and one patient due to paracetamol abuse. Finally, 12 patients were enrolled, eight females and four males. The main demographic patient characteristics are reported in Table 1.

All patients were under treatment with Rivaroxaban for the prevention of thromboembolic events in non-valvular atrial fibrillation. Three patients had additional co-morbidities: one patient had Diabetes type II, one had Chronic Obstructive Pulmonary Disease (COPD), hypertension and hyperthyroidism, and the third patient had COPD. Fifty-seven implants were placed. Six patients received four implants each, three patients received five implants each, and three patients received six implants each. Out of 57 implants, 12 implants were inserted immediately after tooth extractions and received a peri-implant bone graft (Table 2). 

During the surgeries, no major complications were registered, only 3 patients (25%) experienced slight bleeding, controlled within a couple of minutes with compression only. During the postoperative follow-up period, three patients (25%) suffered slight bleeding that is described, according to Bacci (2016) [21], as slight oozing from the wound incision. A mechanical compression with gauzes was applied to control the bleeding. Only one patient required an additional compression with tranexamic acid before having the slight bleeding controlled. No further bleeding events were reported in the following 2 weeks of follow-up. Patients who experienced intra-operative bleeding events were not the same patients who experienced postoperative bleeding episodes. Out of 6 patients experiencing some minor bleeding complications, four patients had bleeding events at the extraction sites. Four patients (33.3%) suffered from swelling in the first week after surgical procedures (Table 3). 

Among the patients suffering from systemic pathologies, only one -who was affected by diabetes, had slight intra-operative bleeding that was easily controlled with no further consequences. No late complications were reported, and the implants that received augmentation did not show any additional complications. No failures were reported in terms of implant mobility and prosthetic replacement, the implant and prosthetic survival rates were both 100% at the 1 year follow up evaluation. No thromboembolic events were reported one year after the 24-h suspension protocol was applied in this patient cohort.

## 4. Discussion

The outcomes from this retrospective study showed, within the limitations of the investigation, that a 24-h discontinuation of Rivaroxaban for those patients who underwent implant placement, peri-implant bone augmentation, and immediate restoration did not increase the risk for perioperative and postoperative bleeding events, nor for thromboembolic complications. Moreover, the clinical outcomes of dental implants were excellent, with a cumulative survival rate of 100%. This means that patients under treatment with DOACs could receive dental implant treatments without any additional risk of implant failure. Even though the results from this study are promising and favorable in terms of implant treatment, several issues still have to be considered when translating the outcomes into daily practice. First, nine out of twelve patients enrolled in this study had no comorbid conditions, whose presence could eventually have had a role in the bleeding events occurrence. In this regard, it should be considered that the presence of co-morbidities and the use of several types of medications could significantly foster the risk of having perioperative bleeding events because of drug–drug interactions. Therefore, this population does not reflect the systemic health condition of the majority of the general population. Second, the three patients who suffered from comorbidities in this study were extremely well-controlled in terms of comorbid pathologies, as they did not have general health status conditions that would eventually raise the risk of having perioperative bleeding events. Third, the decision of whether or not to interrupt the DOACs was taken in agreement with a medical evaluation since the decision could not be taken by a dentist without a treating physician’s advice, based on general medical status, bleeding risk of the surgery procedure, and thromboembolic/hemorrhagic risk as a function of two test evaluations such as CHAD2VASc [23,24] and HAS-BLED [25]. Conversely, the majority of studies in the scientific literature has dogmatically reported the interruption or continuation of the DOACs, as if the choice was only determined by the bleeding risk assessment, instead of being based on a balance between hemorrhagic risks, in case of continuation, and thromboembolic risks, in case of interruption. The use of direct oral anti coagulants for the prevention of thromboembolic events has tremendously increased during the last few years [26], and many clinicians have to manage patients who are under treatment with such medications and who need oral surgery procedures. It can be assumed that patients under treatment with anticoagulants and in need of oral surgery procedures have a higher risk of bleeding events. Therefore, their management should start from an accurate risk assessment [14,20]. The risk of thromboembolic complications in patients that undergo drug interruption should be continuously balanced with the risk of bleeding events. 

Many studies have been conducted with previous anticoagulant medications, such as VKAs. However, most of these studies mainly evaluated the risks associated with multiple tooth extractions; conversely, data on dental implant procedures are rare [12,15,27]. 

In patients under treatment with VKAs, the literature suggested not to discontinue Oral Anticoagulation Therapy (OAT), in case of minor surgical procedures, thereby preventing possible occurrence. In this regard, Walh et al. (2015) [14] have reported that the embolic morbidity risk in patients whose anticoagulation is suspended for oral surgery procedures exceeds that of significant bleeding complications in patients whose anticoagulation is continued. 

Abayon et al. [28] evaluated patients under treatment with Rivaroxaban that continued, partially interrupted or completely interrupted the anticoagulant treatment before dental procedures. They reported that for 1-day suspension of the medication, there was no documented occurrence of thromboembolic events within the 30 days after the procedure, and these outcomes were in accordance with the conclusions of our study.

Since Rivaroxaban does not have an antidote, patients treated with such medication require strict coagulation tests in order to assess the risk of unexpected thrombotic or hemorrhagic complications. 

The Chromogenic test used to evaluate anti-factor Xa activity in plasma is the preferred laboratory test for monitoring the anticoagulant effect of Rivaroxaban. However, its high costs and its unavailability in most laboratories make this monitoring system difficult. In patients with normal renal functions, no routine coagulation test should be required since Rivaroxaban‘s short half-life, and rapid excretion in the urine can guarantee proper management in cases of bleeding complications. Firriolo & Hupp (2012) [7] have indicated that in patients with normal renal function, in treatment with Rivaroxaban and in the absence of any other risk of hemostatic disorders, it is not necessary to interrupt its administration before dental treatments, including simple dental extractions.

Generally, dental procedures with low-bleeding risk (e.g., dental treatments) do not require the suspension of the DOACs in patients with normal renal function. It is suggested that the procedures be performed far from the last dose, i.e., 12 h in case of medications taken twice a day (Apixaban and Dabigatran) or 24 h in case of those taken once a day (Rivaroxaban and Edoxaban). 

On the other hand, in case of procedures at a high risk of bleeding, it is recommended to delay the morning dose of DOACs administered once daily or to skip the morning dose when administered twice daily. 

The pharmacokinetics and pharmacodynamics of DOACs enable the rapid recovery of an anticoagulant effect after complete hemostasis is achieved [29]. 

However, when it is deemed probable that oral or maxillofacial surgery procedures might cause excessive bleeding and/or hemostasis problems, Rivaroxaban should be suspended at least 24 h before the proposed surgery. The risk of hemorrhage will depend on the type and complexity of the surgical procedure, the presence and degree of any kidney impairment, and the presence of other risks for impaired hemostasis. Medication should recommence 24–48 h after surgery.

No bridging therapy is required in patients under treatment with DOACs.

As reported by Beyer-Westerdorf et al. (2014) [20] in their prospective study, patients that went through a short-term suspension of DOACs without heparin bridging did not report any increase of cardiovascular event rates, while major bleeding complications were detected in patients receiving heparin bridging. 

Anyway, each treatment and medical management of the DOACs should always be individualized based on the specific parameters of the patient and the need for concomitant interventions [29]. 

The literature reports very few studies about multiple implant placement performed in patients under treatment with Rivaroxaban [12,27]. Gomez-Moreno et al. (2015) [12] performed up to three multiple implant placements without the suspension of Rivaroxaban, reporting no statistical differences in bleeding complications compared to those in which the medication was suspended for 48 h. Clemm et al. (2016) [27] placed up to 10 implants in patients taking anticoagulant medications without interruption of the administration and reported no major postoperative bleedings. However, this latter study does not specify if the ten implants were placed in a patient taking Rivaroxaban or a different Oral Anticoagulant Medication (OAM). 

When managing these patients, there is an important need for proper classification of oral surgical interventions. In the current literature, as shown by Kammerer et al. (2015) [13], the term “minor oral surgery” is widely used to describe any procedures performed by dentists. This misconception may lead to an underestimation of the risks when these kinds of patients must undergo complex oral procedures. On the other hand, the Scottish dental guidelines [30] have suggested interruption of DOACs in case of high bleeding risk procedures, such as more than three tooth extractions or more than two implant placements. Therefore, to follow the above-reported suggestions, to reduce the steps necessary to complete the whole implant treatment, and upon the physician’s advice, we planned to interrupt the medication for 24 h and to place and restore implants simultaneously to reduce to the utmost the number of events that could have put patients at risk of bleeding events. Our treatment strategy was supported by many studies on immediate implants and immediate loading. Covani et al. [19] reported a cumulative survival rate of 95.1%, ensuring satisfying clinical outcomes. In this cohort, Rivaroxaban was taken once daily with the evening meal. Therefore, skipping the dose intake the night before the surgery and restarting it the same day of the surgery could guarantee to the surgeon a therapeutic window of 24 h in which the bleeding events and complications could be reduced.

The results of this study led to an intra- and post-operative bleeding rate of 25%, slightly higher than the outcomes reported in the literature [12]. However, all the bleeding events were minor ones, easily controlled within a couple of minutes, except for one case, and it did not negatively influence the final outcome of the treatment. These outcomes should be cautiously considered due to the low number of participants, as well as the absence of significant co-morbidities and drug intake that, if present, could have had a notable impact on the bleeding event occurrence.

The retrospective design and the limited cohort of patients account for the major limitations of this study. However, this could be a starting point and could endorse larger and prospective studies. The fact that every patient was medically well-managed is favorable for eliminating a confounding factor. However, this could be a limit when translating the outcomes of this investigation.

## 5. Conclusions

Accurate management of anticoagulated patients may guarantee the possibility to perform implant surgery with an immediate prosthesis delivery safely. Patients taking DOACs may present complex medical status. Thus, the importance of collaborative communication between the surgeon and the patient’s physician remains fundamental. Within the limitations of this retrospective study, multiple implant placements with an immediate loading can be performed without any significant bleeding complications with a 24 h discontinuation of Rivaroxaban following the advice of the patient’s treating physician.

## Figures and Tables

**Table 1:** Description of the samples.

Gender	Male	Female
No. of patients	4	8
Mean age at the time of recruitment	65.1 ± 8.57	
Range	50–81	
**Smoking Habit**	**Yes**	**No**
No. of patients	3	9
**Presence of Multiple Diseases**	**Yes**	**No**
No. of patients	3	9
Patient 1	Diabetes	
Patient 2	COPD, Hypertension, Hyperthyroidism	
Patient 3	COPD	

**Table 2:** Implant distribution.

No. of Implants Supporting Fixed Prosthesis	No. of Patients	Patients with Comorbidities	Tot. Implants Placed (*Post-Ex*)
4	6	1 (Diabetes)	24 (*8*)
5	3	1 (COPD, Hypertension, Hyperthyroidism)	15 (*0*)
6	3	1 (COPD)	18 (*4*)
			57 (*12*)

**Table 3:** Description and incidence of bleeding events on the twelve (12) patients.

Surgical Time	No Bleeding	Slight Bleeding	Moderate Bleeding	Severe Bleeding	Healing Complications (Edema, Swelling, Hematoma)
Intra-operative	9 (75%)	3 (25%)	0	0	-
Post- operative	9 (75%)	3 (25%)	0	0	4 (33.3%) (swelling)
